# Factor VIII-Fc Activates Natural Killer Cells *via* Fc-Mediated Interactions With CD16

**DOI:** 10.3389/fimmu.2021.692157

**Published:** 2021-06-28

**Authors:** H.A. Daniel Lagassé, Louis B. Hopkins, Wojciech Jankowski, Marc G. Jacquemin, Zuben E. Sauna, Basil Golding

**Affiliations:** ^1^ Hemostasis Branch, Division of Plasma Protein Therapeutics, Office of Tissues and Advanced Therapies, Center for Biologics Evaluation and Research, Food and Drug Administration, Silver Spring, MD, United States; ^2^ Department of Cardiovascular Sciences, Center for Molecular and Vascular Biology, University of Leuven, Leuven, Belgium

**Keywords:** Fc-fusion, immunogenicity, Fc gamma receptors, natural killer cells, antibody-dependent cellular cytotoxicity

## Abstract

The most challenging complication associated with Factor VIII (FVIII) replacement therapy is the development of neutralizing anti-drug antibodies, or inhibitors, which occur in 23-35% of severe (FVIII level <1%) hemophilia A (HA) patients and are a serious hindrance to effective management of HA. Consequently, strategies that can either prevent anti-FVIII inhibitors from developing or “tolerize” individuals who develop such antibodies represent a clinically important unmet need. One intervention for patients with high-titer inhibitors is immune tolerance induction (ITI) therapy. Although ITI therapy is the only clinically proven strategy to eradicate anti-FVIII inhibitors, mechanisms of inhibitor reduction remain unknown. Factor VIII Fc-fusion (rFVIIIFc) is an enhanced half-life antihemophilic factor used in replacement therapy for HA. Fc-fusion is a successful protein bio-engineering platform technology. In addition to enhancement of plasma half-life *via* neonatal Fc receptor (FcRn) binding, other Fc-mediated interactions, including engagement with Fc gamma receptors (FcγR), may have immunological consequences. Several case reports and retrospective analyses suggest that rFVIIIFc offers superior outcomes with respect to ITI compared to other FVIII products. Previously we and others demonstrated rFVIIIFc interactions with activating FcγRIIIA/CD16. Here, we investigated if rFVIIIFc activates natural killer (NK) cells *via* CD16. We demonstrated rFVIIIFc signaling *via* CD16 independent of Von Willebrand Factor (VWF):FVIII complex formation. We established that rFVIIIFc potently activated NK cells in a CD16-dependent fashion resulting in IFNγ secretion and cytolytic perforin and granzyme B release. We also demonstrated an association between rFVIIIFc-mediated NK cell IFNγ secretion levels and the high-affinity (158V) CD16 genotype. Furthermore, we show that rFVIIIFc-activated CD16^+^ NK cells were able to lyse a B-cell clone (BO2C11) bearing an anti-FVIII B-cell receptor in an antibody-dependent cellular cytotoxicity (ADCC) assay. These *in vitro* findings provide an underlying molecular mechanism that may help explain clinical case reports and retrospective studies suggesting rFVIIIFc may be more effective in tolerizing HA patients with anti-FVIII inhibitors compared to FVIII not linked to Fc. Our *in vitro* findings suggest a potential use of Fc-fusion proteins acting *via* NK cells to target antigen-specific B-cells, in the management of unwanted immune responses directed against immunogenic self-antigens or therapeutic protein products.

## Introduction

Hemophilia A (HA) is a genetic disorder caused by a deficiency in functional Factor VIII (FVIII) levels. FVIII replacement therapy is used to treat HA. Several novel bioengineered FVIII therapeutic protein products have been approved in the last decade. One of these is a recombinant FVIII Fc-fusion protein (rFVIIIFc) designed to enhance the plasma half-life of FVIII. The most challenging complication associated with FVIII replacement therapy is the development of neutralizing anti-drug antibodies, or inhibitors, which occur in 23-35% of severe (FVIII level <1%) HA patients and are a serious hindrance to the effective management of hemophilia A (1, 2). Consequently, strategies that can either prevent anti-FVIII antibodies from developing or “tolerize” individuals who develop such antibodies represent a clinically important unmet need. The clinical intervention for patients with high-titers of inhibitors is immune tolerance induction (ITI); i.e. high-dose FVIII infused over several months to tolerize the immune system. Depending on the protocol, studies have shown that ITI is successful in 50% to 88% of patients (3, 4). The success rate for “rescue ITI” (use of an alternative product for those who have failed an initial ITI regimen) is much lower. Several retrospective studies and case reports (5–7) have shown that rFVIIIFc, (i) has a lower median time to tolerization and (ii) is often successful in rescue ITI. However, these data sets are small and not conclusive. Although ITI therapy is the only clinically proven strategy to eradicate anti-FVIII inhibitors, mechanisms of inhibitor reduction remain unknown.

Therapeutic Fc-fusion protein products have been used to successfully treat many diseases (8–10). An Fc-fusion protein used as a drug consists of an immunoglobin Fc-domain linked to a bioactive protein or peptide which provides the pharmacological effect. The Fc-domain affects the biophysical and biochemical properties of the active moiety, making it a better therapeutic (11). A common impetus for developing an Fc-fusion protein is to increase the plasma half-life *via* interaction of the Fc with the neonatal Fc-receptor (FcRn). Following endocytosis, Fc-fusion proteins bind to FcRn located within acidified endosomes where they are recycled instead of continuing down a catabolic pathway (12). Additionally, the larger size of the fusion-protein can lead to slower renal clearance (13). Finally, the Fc-domain allows affinity purification using Protein A or Protein G, simplifying the manufacturing process and providing cost savings (14). Due to these advantages, 15 Fc-fusion products have been approved by the U.S. FDA and many more are in the pipeline.

Besides recycling of the protein mediated by Fc-FcRn, the IgG1 Fc-domain also engages with several canonical and non-canonical Fc-receptors (FcRs) (15, 16). These FcRs play important roles in many immunological responses (16, 17). The FcR relevant to this study is Fc gamma receptor IIIA (FcγRIIIA/CD16), an activating FcγR, with low to medium affinity for IgG1 Fc; primarily expressed by monocytes, macrophages and natural killer (NK) cells (18). The binding of the IgG1 Fc-domain to CD16 on NK cells combines the specificity of antibodies to the potent effector function of NK cells through a phenomenon called ADCC (19). Several clinically used monoclonal antibodies (mAbs) (e.g. trastuzumab and rituximab) specifically destroy cancer cells by exploiting ADCC (20, 21). These therapeutic mAbs bind to molecules expressed on the target cells with their antigen binding (Fab) regions and to NK cells *via* the Fc-FcγRIIIA interaction. When fused to the IgG1 Fc-domain a protein antigen that binds to a cell surface receptor on a specific cell can, in principle, target and lyse that cell *via* ADCC. An example is a protein antigen that specifically binds to the B-cell receptors (BCR) on a B-cell while the Fc moiety engages with CD16 on NK cells. Such targeted destruction of memory B-cells could provide a tool for controlling anti-drug antibody immune responses to therapeutic proteins.

We previously reported the unexpected observation that monomeric rFVIIIFc bound and signaled *via* CD16 using BW5147:hCD16ζ reporter cells (22). Thus, we hypothesized that specific killing of memory B-cells expressing anti-FVIII BCR by CD16^+^ NK cells and mediated by rFVIIIFc could prove a mechanistic explanation for the recent clinical findings vis-à-vis ITI. In this study, we investigated the hypothesis that rFVIIIFc mediates selective destruction of memory B-cells that express anti-FVIII antibodies. We demonstrate that rFVIIIFc engages with CD16, activates CD16^+^ NK cells, and induces NK cell-mediated cellular cytotoxicity of an anti-FVIII B-cell clone from an inhibitor-positive hemophilia A patient.

## Materials and Methods

### Materials

rFVIIIFc and rFIXFc were acquired from Biogen through a Material Transfer Agreement. Biogen provided rFVIIIFc protein (1.02 mg/mL; 4.6 µM). The rFVIIIFc protein was adjusted to the final working concentration using assay buffer and was used to achieve hyper-physiological FVIII levels in *in vitro* assays. rFVIIIFc drug product (DP) [ELOCTATE, 2270 IU vial] and rFVIII [XYNTHA, 3120 IU Solofuse syringe] were purchased from ASD Healthcare. Anti-CD20 [rituximab] and anti-CD20 [obinutuzumab] were acquired from the NIH Pharmacy. NISTmAb (RM 8671), Humanized IgG1κ Monoclonal Antibody was purchased from the National Institute of Standards and Technology. The anti-CD20 monoclonal antibodies, rFIXFc, rFVIIIFc DP and rFVIII were reconstituted with manufacturer-provided diluent per manufacturer’s instructions. The reconstituted drug products [rituximab, 10 mg/mL (69 µM); obinutuzumab, 25 mg/mL (167.5 µM); rFIXFc, 1 mg/mL (10.2 µM); rFVIIIFc DP, 100.9 µg/mL (458.6 nM); rFVIII, 101.3 µg/mL (595.9 nM)] were then adjusted to the final working concentration using assay buffer. All therapeutic proteins were frozen at -80°C as small aliquots and thawed on ice prior to use. Blocking F(ab’)_2_ fragments [anti-human CD64 (FcγRI) Clone 10.1; anti-human CD32 (FcγRII) Clone 7.3; anti-human CD16 (FcγRIII) Clone 3G8] were purchased from Ancell.

NK-92 (ATCC #CRL-2407) and PTA-6967 (ATCC #PTA-6967) were maintained in Alpha Minimum Essential medium without ribonucleosides and deoxyribonucleosides but with 2 mM L-glutamine and 1.5 g/L sodium bicarbonate (Gibco #12561-056) and supplemented with 0.2 mM inositol, 0.1 mM 2-mercaptoethanol, 0.02 mM folic acid, 12.5% horse serum (ATCC #30-2040), 12.5% fetal calf serum (Gibco #10438-034), and 100 U/mL recombinant IL-2 (R&D Systems #202-IL), herein referred to as NK cell media.

BO2C11, a human lymphoblastoid cell line was generated from an inhibitor positive hemophilia A patient using the Epstein-Barr virus (23). Raji (ATCC #CCL-86) and BO2C11 were maintained in RPMI 1640 media supplemented with GlutaMAX (Gibco #35050-061) and 10% fetal calf serum (Gibco #10438-034).

### ADCC Reporter Bioassay

The ADCC Reporter Bioassay (Promega #G7015) is a surrogate assay for monitoring cellular cytotoxicity by NK cells mediated by Fc engagement with CD16. In this assay the effector cells, Jurkat cells, are engineered to express CD16 (with the V158 polymorphism) on their surface and contain a reporter gene, nuclear factor of activated T cell response element (NFAT-RE) upstream of luciferase (24). In some experiments, (i) target (Raji) cells were not included or (ii) the manufacturer provided RPMI 1640 media was replaced with AIM-V serum free media ± 2 nM VWF (Haemotologic Technologies #HCVWF-0191) (VWF concentration equivalent to ~5% serum) or (iii) the manufacturer provided low IgG fetal calf serum was replaced with normal human serum (Assaypro #UD203011) or with VWF-deficient human serum (Assaypro #D203011).

### NK Stimulation Assay

In 96-well V-bottom cell culture plates, NK-92 or PTA-6967 cells (100,000/well) were incubated overnight at 37°C, 5% CO_2_ in NK cell media containing therapeutic protein products, polyclonal human IgG, or PMA (50 ng/mL) and ionomycin (1 µg/mL). For blocking studies, NK cells were pre-bound with FcγR-specific F(ab’)_2_ fragments (10 µg/mL) prior to the addition of rFVIIIFc. Following incubation, NK cells were pelleted, and cell culture supernatants were tested for human IFNγ (BD #555142), granzyme B (Invitrogen #BMS2027), and perforin (Invitrogen #BMS2306) levels by sandwich ELISA.

### NK Cell Isolation

Cryopreserved human PBMCs were purchased from Cellular Technology Limited. Human NK cells were isolated from PBMCs using Miltenyi NK Cell Isolation Kit (Miltenyi #130-092-657) with MS Columns (Miltenyi #130-042-201), and a MiniMACS Separator (Miltenyi #130-042-102). Stimulation of primary human PBMC and NK cell fractions were performed as described above.

### NK Cell Degranulation Assay

In 12-well cell culture plates, PTA-6967 cells (1,000,000/well) were incubated for 6 hours at 37°C, 5% CO_2_ in NK cell media containing 5 µL anti-CD107a-APC (BD #560664) as well as PMA (50 ng/mL) and ionomycin (1 µg/mL), or rFVIIIFc (250 nM). After one hour, monensin (0.67 µL/mL) and Brefeldin A (1 µL/mL) were added to block endocytic trafficking. Cell samples were harvested, washed and stained for viability (Fixable Viability Dye eFluor506; eBioscience #65-2860) and surface markers (CD3-FITC (BD #561806), CD16-PE-Cy5 (BD #555408), CD56-PE-Cy5 (BD #557747)). Samples were analyzed using a BD LSRII cytometer and FlowJo version 10 software.

### FVIII-Specific B-Cell Killing Assay (Modified ADCC)

In a modified ADCC assay, target BO2C11 B-cells were prepared in assay buffer (RPMI-1640 media with 5% fetal calf serum) and added (20,000 cells/well) to a V-bottom 96 well plate. rFVIIIFc was prepared to appropriate concentrations in assay buffer and added to target B-cells. Effector NK cells (CD16^-^ NK-92 or CD16^+^ PTA-6967) were prepared in assay buffer and added (100,000 cells/well) to the target cells and rFVIIIFc. Target cells lysed with 1% Triton X-100 served as maximal-lysis (positive) controls. Wells containing no rFVIIIFc served as spontaneous-lysis (negative) controls. The 96-well culture plates were incubated 4 hours at 37°C, 5% CO_2_. Following incubation, the cells were pelleted, and the cell culture supernatants were transferred to another 96-well plate for further analysis. Lactate dehydrogenase (LDH) release was measured using CytoTox 96 Non-Radioreactive Cytotoxicity Assay according to manufacturer’s instructions (Promega #G1782).

## Results

### rFVIIIFc Signals *via* CD16

**Figure 1:**
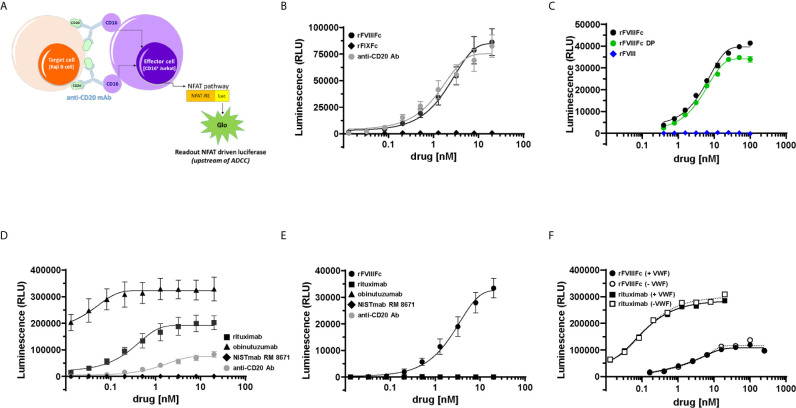
rFVIIIFc signals *via* CD16. **(A)** An illustration depicting the ADCC Reporter Bioassay used to measure CD16-mediated responses to human IgG1 Fc-containing therapeutic protein products or control antibodies. **(B)** ADCC Bioassay Target Cells (1.25 x 10^4^ CD20^+^ Raji B-cells) were incubated with a dilution series (20 nM – 13.1 pM) of therapeutic Fc-fusion proteins (rFVIIIFc, black circles; rFIXFc, black diamonds) or anti-CD20 assay control antibody (grey circles) and then incubated with ADCC Bioassay Effector Cells (7.5 x 10^4^) for 6 hours at 37°C. Following incubation, Bio-Glo Luciferase Assay Reagent was added, and luminescence was determined using a Perkin Elmer Victor X3 2030 plate reader. Plotted as mean relative luminescence units (RLU) ± standard error of mean (SEM) (n = 2-3) from two independent experiments. **(C)** ADCC Reporter Bioassay conducted as described above with dilution series (100 nM – 391 pM) of rFVIIIFc protein (black circles), rFVIIIFc DP (green circles), or rFVIII (blue diamonds); plotted as mean ± SEM (n = 2-4) from a representative of two experiments. **(D)** ADCC Reporter Bioassay conducted as described above with monoclonal antibodies as controls (rituximab, black squares; obinutuzumab, black triangles; NISTmAb RM 8671, black diamonds; anti-CD20 assay control antibody, grey circles); plotted as mean ± SEM (n=2-3) from two independent experiments. **(E)** ADCC Reporter Bioassay conducted as described above, except in the absence of ADCC Bioassay Target Cells (rFVIIIFc, black circles; rituximab, black squares; obinutuzumab, black triangles; NISTmAb RM 8671, black diamonds; anti-CD20 assay control antibody, grey circles); plotted as mean ± SEM (n = 2) from a representative of two experiments. **(F)** ADCC Reporter Bioassay experiment conducted as described above, except assay buffer constituents RPMI 1640 Medium and 4% Low IgG fetal calf serum were replaced with AIM-V serum free medium + 2 nM VWF (solid lines/closed symbols) or AIM-V serum free medium (dashed lines/open symbols) (rFVIIIFc, circles; rituximab, squares); plotted as data points (n = 1) from a representative of two experiments.

Previously we had shown that monomeric rFVIIIFc and recombinant Factor IX Fc-fusion protein (rFIXFc) bound and signaled *via* CD16 using BW5147:hCD16ζ reporter cells (22). To determine whether these findings translated into activation of CD16^+^ NK cell function, we employed the ADCC Reporter Bioassay system (24). We tested rFVIIIFc, rFIXFc and anti-CD20 mAb (positive-control) for stimulation of CD16-mediated signaling. The luciferase signal generated by CD16-activated effector cells provides a surrogate endpoint for NK cell mediated-cellular cytotoxic responses (**Figure 1A**). When co-incubated with effector and target cells (Raji B-cells expressing the surface antigen CD20), rFVIIIFc stimulated CD16^+^ effector cells to produce a luciferase signal to a similar degree as an assay-specific positive control anti-CD20 mAb. However, rFIXFc did not elicit a CD16-mediated signaling response (**Figure 1B**), despite having the identical Fc primary amino acid sequence, hinge region, protein expression system, and manufacturer as rFVIIIFc. We observed no difference in CD16 signaling by rFVIIIFc protein and rFVIIIFc DP samples in the ADCC Reporter Bioassay (**Figure 1C**). A beta-domain deleted rFVIII therapeutic did not elicit a response suggesting rFVIIIFc’s IgG1 Fc domain is responsible for the observed CD16 signaling, not the rFVIII moiety. Two B-cell targeting (anti-CD20) therapeutic mAbs (rituximab and obinutuzumab) were used as additional positive controls (**Figure 1D**), as their mechanisms of action involves ADCC (25, 26). A non-B-cell targeting mAb (NISTmAb RM 8671) was used as a negative control and exhibited no luciferase response from the CD16^+^ effector cells (**Figure 1D**).

Since FcγRIIIA/CD16 is a low-affinity receptor for human immunoglobulin G1 (IgG1), Fc-multimerization and receptor crosslinking is a prerequisite for signaling *via* CD16 (27). For IgG1 Fc-containing proteins, protein aggregates (Fc-multimerization) could trigger FcγR signaling. Thus, we assessed the aggregate level of the rFVIIIFc samples used in our studies by non-reducing SDS-PAGE (data not shown) and size exclusion chromatography, which revealed no evidence of rFVIIIFc aggregates (**Supplementary Figure 1**). In the case of anti-CD20 mAbs, Fab engagement with CD20 on the target cell surface allows Fc multimerization, CD16 receptor crosslinking, and effector cell activation (28). Consistent with this postulate, none of the three anti-CD20 mAbs tested elicited a luciferase signaling response in the absence of CD20^+^ Raji cells (**Figure 1E**). However, we observed rFVIIIFc stimulation of CD16^+^ effector cells in the absence of Raji target cells (**Figure 1E**) suggesting that rFVIIIFc-CD16 signaling does not require target cell-binding and/or multimerization.

We hypothesized that rFVIIIFc elicits CD16 signaling because, under physiological conditions, FVIII molecules are associated with circulating VWF multimers (29). Thus, the VWF present in fetal bovine serum (FBS) used in the assay buffer could provide a scaffold for Fc multimerization on the NK cell and subsequent CD16 signaling. To test this hypothesis, we performed the ADCC assay with rFVIIIFc in the presence and absence of human VWF. When tested in AIM-V serum free medium, rFVIIIFc continued to stimulate luciferase activity in the CD16^+^ effector cells. We observed no difference in the rFVIIIFc-CD16 signaling response when serum-free medium was spiked with VWF (2 nM; equivalent to ~5% final serum concentration) (**Figure 1F**); as expected, the absence of VWF had no effect on rituximab-mediated CD16 signaling (**Figure 1F**). Furthermore, we also observed no effect of rFVIIIFc-mediated CD16 signaling when the ADCC Reporter Bioassay was performed using RPMI 1640 medium containing VWF-deficient human serum as compared to normal human serum (**Supplementary Figure 2**). These findings suggest that rFVIIIFc-mediated CD16 signaling is independent of VWF : FVIIIFc multimerization. Unlike rFVIIIFc, rFIXFc and rituximab in the absence of B-cell targets fail to activate the CD16^+^ effector cells, suggesting that the FVIII component of rFVIIIFc may play a role in enhancing the Fc-FcγRIIIA interaction.

### rFVIIIFc Activates and Induces Degranulation and IFNγ Secretion by CD16^+^ NK Cells

**Figure 2:**
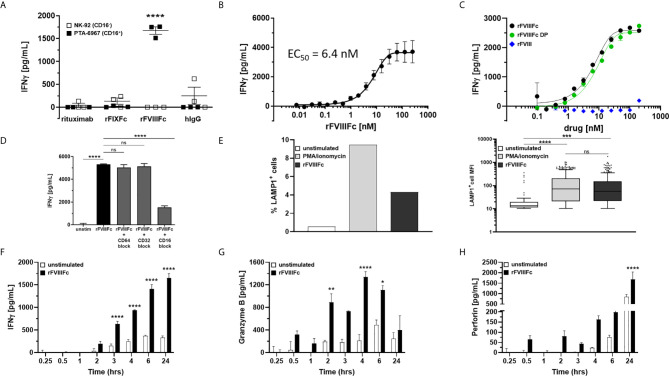
rFVIIIFc activates and induces degranulation by CD16^+^ NK cells. **(A)** IFNγ secretion by CD16^-^ NK cells (NK-92; open squares) or CD16^+^ NK cells (PTA-6967; filled squares) measured by ELISA following overnight incubation with 67 nM human IgG1 Fc-containing proteins [rituximab; rFIXFc; rFVIIIFc; human polyclonal IgG]; plotted as mean ± SEM (n=3) from a representative of two experiments. Tukey’s multiple comparisons tests (two-tailed) were performed for each protein between stimulated CD16^-^ NK cells (open squares) and stimulated CD16^+^ NK cells (filled squares). ****p < 0.0001. **(B)** Secreted IFNγ response of CD16^+^ NK cells (PTA-6967) following overnight stimulation with different concentrations of rFVIIIFc (250 nM to 7.6 pM); plotted as mean ± SEM (n = 6) from 2 independent experiments. **(C)** Secreted IFNγ response of CD16^+^ NK cells (PTA-6967) as above with rFVIIIFc protein (black circle), rFVIIIFc DP (green circle), or rFVIII (blue diamond) (200 nM to 98 pM); plotted as mean ± SEM (n = 2) from a representative of two experiments. **(D)** IFNγ secretion by CD16^+^ NK cells (PTA-6967) stimulated with rFVIIIFc (250 nM). For FcγR blockade, CD16^+^ NK cells were pre-incubated with FcγR-specific F(ab’)_2_ fragments prior to stimulation with rFVIIIFc; plotted as mean ± SEM (n = 4). Tukey’s multiple comparisons tests (two-tailed) were performed between (i) unstimulated controls and rFVIIIFc stimulated and (ii) rFVIIIFc stimulated ± FcγR blockade. ****p < 0.0001. **(E)** Left, bar graph depicting surface CD107a (LAMP1) levels (% LAMP1^+^ cells), an indirect measure of degranulation, measured on NK cells (PTA-6967) from a single flow cytometry experiment following a 6-hour stimulation with PMA/ionomycin (50 ng/mL PMA, 1 µg/mL ionomycin) or rFVIIIFc (250 nM); right, Tukey box-and-whiskers plots depicting LAMP1^+^ cell mean fluorescence intensity (MFI). One-way ANOVA and Tukey’s multiple comparisons test performed, ***p = 0.0004, ****p < 0.0001. **(F–H)** Measurement of secreted IFNγ **(F)**, granzyme B **(G)**, and perforin **(H)** by ELISA over time from rFVIIIFc-stimulated (67 nM) CD16^+^ NK cells (PTA-6967) plotted as mean ± SEM (n = 3). Tukey’s multiple comparisons tests (two-tailed) were performed at each timepoint between unstimulated cells (open bars) and rFVIIIFc stimulated cells (filled bars). *p < 0.05, **p < 0.01, ****p < 0.0001. ns, not significant, p > 0.05.

Although we demonstrate engagement of rFVIIIFc with CD16, and signaling *via* a reporter-gene, this does not necessarily mean NK cells are fully activated leading to granzyme and perforin release and cytokine (IFNγ) secretion. To determine whether rFVIIIFc fully activated NK cells *via* CD16 we used two NK cell lines; NK-92 (which is CD16^-^) and PTA-6967 (which is CD16^+^). The CD16^+^ NK cell line was stimulated by rFVIIIFc to secrete IFNγ, but not by any other Fc-fusion protein or antibody tested (**Figure 2A**). Even upon plate-immobilization of the Fc-containing proteins, mimicking aggregation or immune complex formation to enhance CD16 crosslinking, rFIXFc failed to stimulate IFNγ secretion by CD16^+^ NK cells (**Supplementary Figure 3**). This is consistent with a lack of rFIXFc activity in the ADCC Reporter Bioassay (**Figure 1B**). Importantly, none of the therapeutic proteins tested (including rFVIIIFc) stimulated IFNγ secretion from the CD16^-^ NK cell line (**Figure 2A**, open squares). These results confirmed rFVIIIFc can activate NK cells in a CD16-dependent fashion. IFNγ secretion mediated by rFVIIIFc is dose dependent with an EC_50_ of 6.4 nM (**Figure 2B**). Consistent with results shown in **Figure 1C**, we observed no difference between rFVIIIFc protein and rFVIIIFc DP in terms of inducing IFNγ secretion from CD16^+^ NK cells, while rFVIII (without the Fc moiety) did not elicit a response (**Figure 2C**). The specificity of the rFVIIIFc : CD16 interaction was further demonstrated by pre-incubation of NK cells with αCD16 F(ab’)_2_ that completely blocked rFVIIIFc-induced IFNγ secretion by NK cells (**Figure 2D**). Pre-incubation of the NK cells with αCD64 F(ab’)_2_ or αCD32 F(ab’)_2_ fragments did not block their activation by rFVIIIFc (**Figure 2D**). This is consistent with our findings using the CD16^-^ NK cell line (**Figure 2A**). We confirmed the surface FcγR expression profiles of NK-92 and PTA-6967 NK cells by flow cytometry (**Supplementary Figure 4**). Moreover, as observed in the ADCC Reporter Bioassay (**Figure 1F** and **Supplementary Figure 2**), the induction of CD16^+^ NK cell IFNγ secretion was not dependent on VWF (**Supplementary Figure 5**).

Elevated surface expression of Lysosomal Associated Membrane Protein 1 (LAMP-1; CD107a) is an indirect measure of NK cell activation and the release of cytolytic granules (15). Flow cytometric analysis of NK cells (PTA-6967) showed that, compared to untreated cells, there was an almost 8-fold increase in the percentage of cells with an elevated level of LAMP1 following a 6-hour treatment with rFVIIIFc (**Figure 2E**). Treatment with PMA and ionomycin, as a positive control for CD16-independent stimulation showed an approximately 17-fold increase in the percentage of cells with elevated LAMP-1 (**Figure 2E**).

Upon activation NK cells secrete cytokines and release granules containing cytolytic proteins (30). IFNγ secretion occurs as early as three hours and continues to increase for 24 hours following rFVIIIFc stimulation (**Figure 2F**). Release of the cytolytic granule proteins granzyme B (**Figure 2G**) and perforin (**Figure 2H**), followed different kinetics compared to the IFNγ response. As a CD16-independent positive control for NK cell activation, we used PMA and ionomycin and observed IFNγ secretion as well as granzyme B and perforin release from CD16^+^ NK cells (**Supplementary Figure 6**).

These findings (**Figures 1** and **2**) indicate that monomeric rFVIIIFc signals *via* CD16 and results in NK cell activation.

### rFVIIIFc Induces IFNγ Secretion From NK Cells Isolated From Human PBMCs

**Figure 3:**
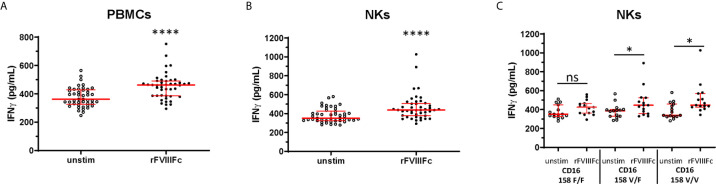
rFVIIIFc induces IFNγ secretion from peripheral blood mononuclear cells and NKs isolated from healthy human donors. IFNγ secretion from PBMCs **(A)** or resting NK cells **(B)** isolated from 15 healthy human donors each measured in triplicate by ELISA following overnight stimulation with rFVIIIFc (250 nM). **(C)** IFNγ secretion from resting NK cells with distinct CD16 V/F 158 genotypes (CD16 158 F/F, V/F, V/V; n = 5, each measured in triplicate). **(A–C)** Median ± inter-quartile range (IQR) indicated in red. Wilcoxon signed rank tests (two-tailed) were performed for comparisons between unstimulated and rFVIIIFc-stimulated cells. *p < 0.05, ****p < 0.0001. ns, not significant, p > 0.05.

In addition to NK cell lines, we assessed the effect of rFVIIIFc stimulation on primary human NK cells isolated from peripheral blood mononuclear cells (PBMCs) collected from 15 healthy donors. We observed significantly (p < 0.0001) elevated IFNγ secretion from human PBMCs as well as isolated NK cells (CD3^-^ CD56^+^) confirmed for surface CD16 expression (**Supplementary Figure 7**) and PMA/ionomycin responsiveness (**Supplementary Figure 8**) following incubation with rFVIIIFc (**Figures 3A, B**). The FCGR3A gene single nucleotide polymorphism (SNP) (rs396991) results in two FcγIIIRA allotypes, FcγIIIRA 158V and FcγIIIRA 158F (31). FcγIIIRA 158V has been demonstrated to bind IgG1 with higher affinity than FcγIIIRA 158F (31) and increased clinical responses to monoclonal antibodies with ADCC-mediated mechanisms of action (32). A subset analysis showed that elevation in secreted IFNγ by NK cells following treatment with rFVIIIFc was significant (p < 0.05) with cells from donors possessing at least one high affinity CD16 158V allele, and not significant with cells from donors with the low affinity 158F/F variant (**Figure 3C**).

### rFVIIIFc Induces BO2C11 (FVIII-Specific B-Cell) Lysis Through Interactions With CD16^+^ NK Cells

**Figure 4:**
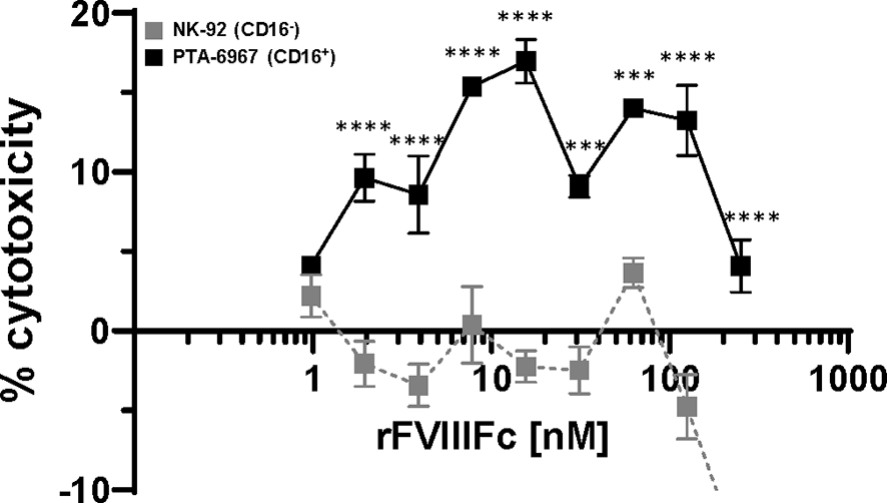
rFVIIIFc induces BO2C11 (FVIII-specific B-cell) lysis through interactions with CD16^+^ NK cells. Lactate dehydrogenase (LDH) release from target BO2C11 B-cells following 4 hour incubation at 37°C following incubation with rFVIIIFc (250 nM – 977 pM) and CD16^+^ NK cells [PTA-6967, black squares] or CD16^-^ NK cells [NK-92, grey squares] using a 5:1 effector to target ratio (100,000:20,000 cells per well); plotted as mean % cytotoxicity ± SEM (n = 4) from a representative of two experiments. % cytotoxicity was calculated as: % cytotoxicity = [(experimental well – spontaneous lysis control)/(Triton X-100 maximal lysis control – spontaneous lysis control)] * 100. Tukey’s multiple comparisons tests (two-tailed) were performed at each rFVIIIFc concentration comparing responses between CD16^-^ (NK-92) and CD16^+^ (PTA-6967) NK cells. ***p < 0.001, ****p < 0.0001.

We tested the hypothesis that rFVIIIFc-mediated stimulation of NK cells to become effector cells (shown above) can lead to the killing of FVIII-specific B-cells. In such a mechanism the Fc-moiety would engage with CD16 on NK cells while the rFVIII would bind to anti-FVIII BCRs. rFVIIIFc-mediated cell killing of BO2C11, a B-cell clone specific for the FVIII-C2 domain (IgG4 kappa) (23), was dependent on CD16^+^ NK cells (**Figure 4**). The rFVIIIFc-mediated BO2C11 cytotoxicity was dose-dependent with a peak cytolytic response observed at 31.25 nM rFVIIIFc (**Figure 4**). However, at higher rFVIIIFc concentrations, we observed a “hook effect” which has been reported in some ternary complexes (33, 34) (**Figure 4**). Furthermore, we observed BO2C11-mediated enhancement of rFVIIIFc-induced IFNγ secretion by the PTA-6967 cell line (**Supplementary Figure 9**). These results indicate that rFVIIIFc can mediate killing of anti-FVIII producing B-cells.

## Discussion

The extended half-life rFVIIIFc product was designed and developed with the primary goal of increasing the plasma half-life (35, 36). The rFVIIIFc was approved in 2014, and substantial clinical experience with this therapeutic provided in case reports and retrospective analyses (5–7) suggest that rFVIIIFc offers superior outcomes with respect to ITI compared to other FVIII products (37). Currently, several prospective clinical studies including the verITI-8 Study (ClinicalTrials.gov # NCT03093480), the ReITIrate Study (ClinicalTrials.gov # NCT03103542), and the Hemophilia Inhibitor Eradication Trial (ClinicalTrials.gov # NCT04303572) are evaluating the efficacy of rFVIIIFc in ITI regimens. Here, we have investigated the hypothesis that rFVIIIFc could engage with CD16 and drive NK cell-mediated killing of memory B-cells expressing an anti-FVIII BCR. We propose a testable hypothesis that may explain superior outcomes with rFVIIIFc in ITI; *viz.* rFVIIIFc could engage with CD16^+^ NK cells and mediate lysis of anti-FVIII memory B-cells. Here we provide *in vitro* evidence in support of this hypothesis.

Immune tolerance has wide applications in circumventing immune responses to proteins that are used therapeutically (38, 39) as well as in auto-immune diseases (40). However, many currently used tolerogenic approaches result in broad immune suppression, thus putting the patient at risk of infection. In the case of FVIII ITI regimens, the Malmö protocol (41), employs high-dose FVIII product (200 IU/kg daily) administered concomitantly with an immunosuppressive agent [i.e. rituximab; cycloheximide; intravenous immunoglobulin (IVIg)]. There are reports of enhanced FVIII inhibitor eradication following a FVIII ITI regimen in combination with rituximab-mediated B-cell depletion (42, 43). Notably, rituximab does not target antibody-secreting plasma cells as they do not express the CD20 surface antigen, nevertheless depletion of the CD20^+^ memory B-cell pool may be sufficient to eradicate FVIII inhibitors and induce tolerance. However, this approach does not specifically target anti-FVIII B-cells as rituximab also depletes non-FVIII specific B-cells. A highly selective tolerogenic approach that depletes antigen-specific B-cells would avoid undesirable broad-spectrum immunosuppression.

**Figure 5:**
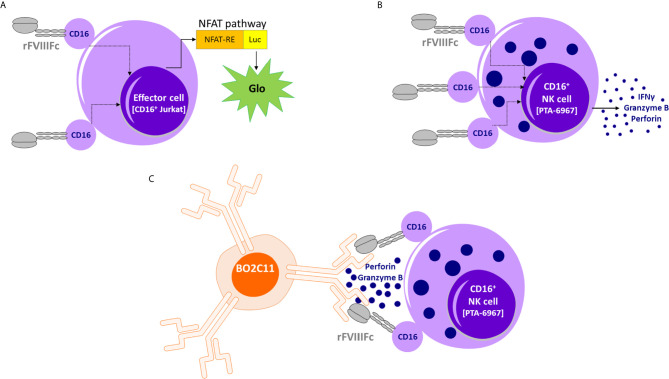
Proposed model of rFVIIIFc : CD16 interactions. **(A)** An illustration depicting interactions between rFVIIIFc and CD16^+^ ADCC Reporter Bioassay effector cells resulting in luciferase activity. **(B)** An illustration depicting interactions between rFVIIIFc and CD16^+^ NK cells leading to IFNγ, granzyme B, and perforin release. **(C)** An illustration depicting interactions between rFVIIIFc and CD16 and a putative mechanism by which rFVIIIFc can target anti-FVIII memory B-cells (BO2C11).

The Fc-domains of antibodies engage with CD16 on the surface of NK cells while the Fab domains provide exquisite selectivity for cellular cytotoxicity by binding to a specific molecule on the target cells (44). Natural non-engineered IgG1 Fc monomers bind to CD16 with low affinity (~ 1 µM) (45). Thus, binding to CD16 at levels that can result in a meaningful biological response requires that the Fc molecules form multimers or complexes thereby elevating the local concentration and resulting in receptor crosslinking (19). For instance, in our ADCC Reporter Bioassay results (**Figure 1**), binding of the anti-CD20 antibodies (e.g. rituximab and obinutuzumab) to target Raji cells is a prerequisite for engagement with CD16^+^ effector cells (**Figure 1E**). In contrast, we observed an exception to this general rule; *viz.* the engagement of rFVIIIFc with CD16^+^ reporter cells was independent of multimerization on the surface of a target cell (**Figures 1E** and **5A**). This is consistent with our previous finding that rFVIIIFc exhibits non-canonical FcγR and complement C1q binding and signaling properties, even in a monomeric form (22). The rFVIIIFc-mediated signals *via* CD16 in these assays could suggest a response driven by protein aggregation or the formation of immune complexes. However, non-reducing SDS-PAGE (data not shown) and size exclusion chromatography analyses of rFVIIIFc used in our studies revealed no evidence of rFVIIIFc aggregates (**Supplementary Figure 1**). Our results provided strong evidence that (unlike other Fc-fusion proteins and mAbs tested) monomeric rFVIIIFc can bind CD16 and stimulate NK cells with relatively high potency (EC_50_ 6.4 nM). This finding is consistent with a previous study demonstrating rFVIIIFc engaged CD16 on the surface of monocyte-derived macrophages (46).

A plausible explanation for the surprising rFVIIIFc signaling in the absence of the prerequisite multimerization is an alternate mechanism for complex formation. Human FVIII has very high affinity for human VWF (0.2 nM) (47), consequently VWF acts as a chaperone for FVIII increasing its circulating half-life from ~2.5 to 12 hours (47, 48). A single FVIII molecule (C2 domain) can bind to the D’D3 domains of a VWF monomer (48). VWF circulates as large multimers which could provide a scaffold for sufficient rFVIIIFc multimerization to foster engagement with CD16. To test the hypothesis that VWF:rFVIIIFc interactions contributed to rFVIIIFc-mediated signaling *via* CD16 we performed the ADCC Reporter Bioassay and *in vitro* NK cell stimulation in the absence and presence of human VWF. We demonstrated rFVIIIFc-mediated CD16 signaling was not abrogated in the absence of VWF (**Figure 1F** and **Supplementary Figures 2, 5**). This suggests that the FVIII component of rFVIIIFc, unlike the FIX of rFIXFc and the Fab of rituximab, enables Fc : CD16 interactions that lead to NK cell activation and effector function.

To determine whether rFVIIIFc : CD16 interactions are sufficient to selectively kill anti-FVIII memory B-cells we used BO2C11 cells. BO2C11 is a human IgG4 kappa B-cell clone with anti-FVIII C2 domain specificity obtained from a hemophilia A subject with high-level inhibitors [for details see (23, 49)]. Using BO2C11 as the target cell in an ADCC assay we demonstrated BO2C11 killing mediated by rFVIIIFc in the presence of CD16^+^ NK cells. Understanding the mechanistic details of rFVIIIFc : CD16 interactions and under what conditions this engagement can be exploited for the killing of FVIII-specific memory B-cells lays the groundwork for the design of future Fc-fusion proteins for use as antigen-specific B-cell targeting agents in immune tolerance. Several groups have initiated efforts to design Fc-fusion proteins as antigen-specific B-cell depletion agents (50–52).

We acknowledge that rFVIIIFc can also engage with other FcγRs on a variety of immune cell types, which may complicate the extrapolation of our findings to mechanisms operating *in vivo*, in a patient. Previous *in vitro* studies have assessed rFVIIIFc’s role on the activation state of macrophages (46), dendritic cells (53) and B-cells (54). We (22) and others (54) have shown the ability of rFVIIIFc to signal *via* the inhibitory FcγRIIB. Additionally, Fc-mediated interactions with FcγRs resulting in antibody-dependent cellular phagocytosis (ADCP) may also be involved in the *in vivo* setting. However, it is possible that several rFVIIIFc : FcγR-mediated interactions may collectively contribute towards the reduced immunogenicity and induced tolerance observed in hemophilia A mice (55). Therefore, *in vivo* hemophilia A mouse model or clinical studies will be needed to test which FcγR^+^ immune cell populations are critical during high-dose rFVIIIFc ITI regimens for inducing tolerance.

The schematic in **Figure 5** provides a putative mechanism by which rFVIIIFc can target anti-FVIII memory B-cells. Our *in vitro* results, and the proposed mechanism could provide a testable hypothesis that may explain reported clinical results wherein ITI using rFVIIIFc was successful in some patients who have failed tolerizing regimens using FVIII products that were not conjugated with Fc (7). As discussed above, the concept of targeting NK cells specifically to memory B-cells that produce undesirable antibodies (to therapeutics and self-antigens) does not find easy clinical applicability due to the low IgG1 Fc : CD16 affinity. However, the fortuitous CD16 engagement by monomeric rFVIIIFc has allowed us to demonstrate that, if the Fc-moiety can be made to engage with CD16 at high affinity, then the strategy of selectively depleting BCR^+^ memory B-cells producing antibodies to a specific antigen is feasible *in vitro*. The biophysics and structural biology of Fc : CD16 binding are quite well understood (56). Thus protein- and glyco-engineering approaches to enhance Fc : CD16 affinities are plausible (57, 58). For instance, the obinutuzumab Fc region was glyco-engineered for higher affinity for CD16 and more potent ADCC activity (25). Future studies with Fc-engineering hold considerable promise in developing a platform-technology for selectively targeting B-cells with undesired BCRs. This approach may be extended to other target cells, such as cancer cells, using Fc-fusion proteins so that the fusion protein is designed to bind a receptor on the target tumor.

## Data Availability Statement

The original contributions presented in the study are included in the article/**Supplementary Material**. Further inquiries can be directed to the corresponding author.

## Author Contributions

HL, ZS, and BG designed the research. MJ provided critical reagents and critically reviewed the manuscript. HL, LH, and WJ performed the research. HL, LH, WJ, ZS, and BG analyzed the data. HL, ZS, and BG wrote the paper. All authors contributed to the article and approved the submitted version.

## Funding

BG and ZS are funded by intramural grants from the U.S. FDA.

## Disclaimer

These findings are an informal communication and represent our best judgment. These comments do not bind or obligate FDA.

## Conflict of Interest

MJ reports grants from Bayer, Takeda, Pfizer and Sobi, outside the submitted work.

The remaining authors declare that the research was conducted in the absence of any commercial or financial relationships that could be construed as a potential conflict of interest.
