# Supplementary material for: Factor VIII-Fc Activates Natural Killer Cells via Fc-Mediated Interactions With CD16
Source: Front Immunol. 2021 Jun 28;12:692157. doi: 10.3389/fimmu.2021.692157 (PMC8273617; doi:10.3389/fimmu.2021.692157)
Supplement: Supplementary file 1 [file DataSheet_1.docx]

Supplementary Material

**Supplementary Figures**


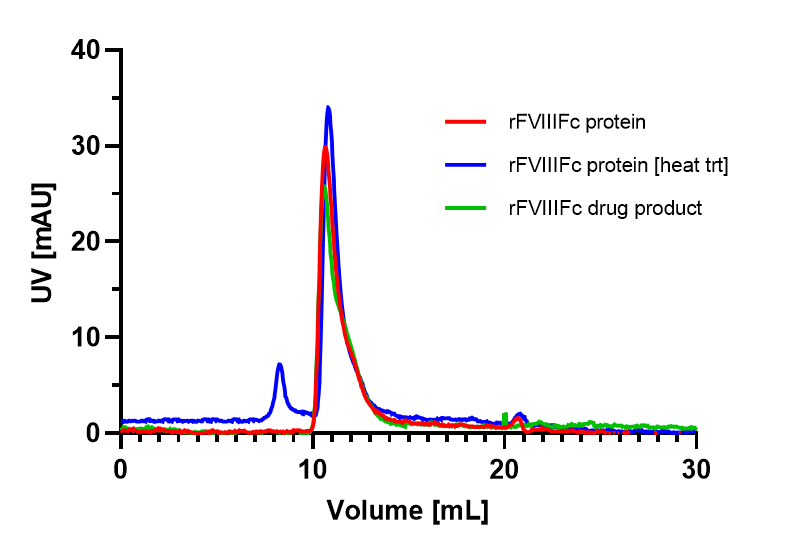


**Supplementary Figure 1.** **Size exclusion chromatography analysis of protein aggregates in rFVIIIFc protein samples**. rFVIIIFc samples (protein or drug product) were thawed on ice, exactly as performed for use in our *in vitro* assays. For size exclusion chromatography analysis, 40 µg of each protein sample was loaded onto a Superdex 200 Increase 10/300 GL column (GE Healthcare) and run on a GE AKTA Purifier 100 Fast Protein Liquid Chromatography (FPLC) system with a flow rate of 0.7 mL/min. As a positive control for aggregate formation, rFVIIIFc protein was subjected to “forced aggregation conditions”, i.e. treating protein sample at 40° C for 24 hours prior to chromatography. Chromatograms depict protein levels detected by UV absorbance (280 nm wavelength) over the collection volume [rFVIIIFc protein, blue; rFVIIIFc drug product, green; rFVIIIFc protein “forced aggregation conditions”, red].


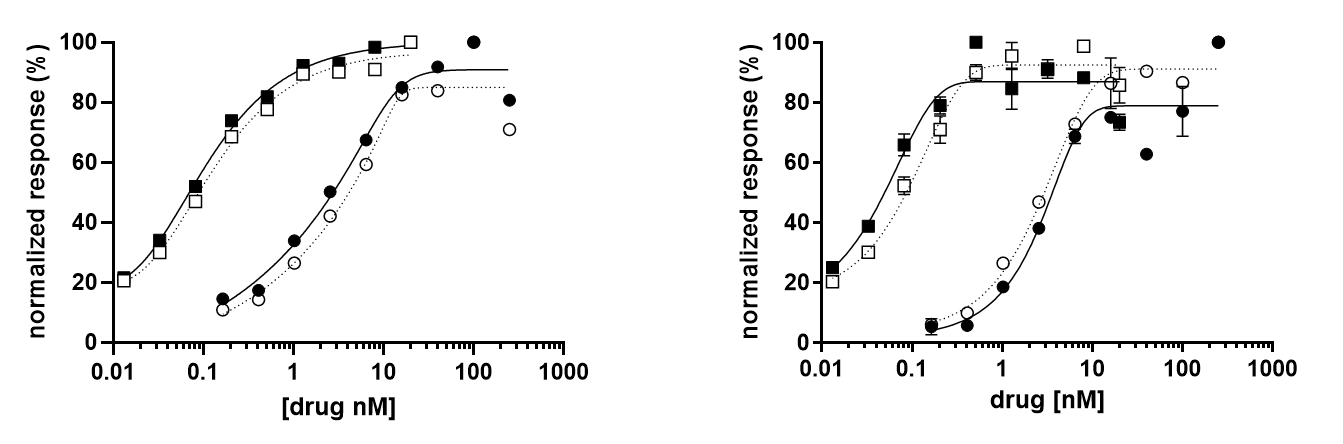


**Supplementary Figure 2.** **rFVIIIFc signals via CD16 in the absence of VWF**. ADCC Bioassay Target Cells (1.25 x 104 CD20+ Raji B cells) were incubated with a dilution series of therapeutic proteins (rFVIIIFc, circles; rituximab, squares) and then incubated with ADCC Bioassay Effector Cells (7.5 x 104) for 6 hrs at 37 °C. Following incubation, Bio-Glo Luciferase Assay Reagent was added, and luminescence was determined using a Perkin Elmer Victor X3 2030 plate reader. The ADCC Reporter Bioassay was conducted according to manufacturer’s instructions, except: (left) assay buffer constituents RPMI 1640 Medium and 4% Low IgG fetal calf serum were replaced with AIM-V serum free medium + 2 nM VWF (solid lines/closed symbols) or AIM-V serum free medium (dashed lines/open symbols); plotted data points from a representative of 2 experiments; (right) the assay buffer constituent 4% Low IgG fetal calf serum was replaced with 4% normal human serum (solid lines/closed symbols) or 4% VWF-deficient human serum (dashed lines/open symbols); plotted as mean ± SEM (n=2) from a representative of 2 experiments. Response levels were normalized to maximal signal for each drug/condition combination.


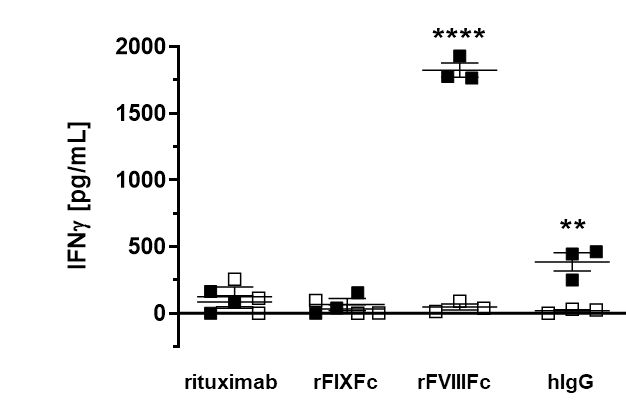


**Supplementary Figure 3.** **Plate-immobilized rFVIIIFc activates CD16^+^ NK cells.** Human IgG1 Fc-containing proteins [rituximab; rFIXFc; rFVIIIFc; human polyclonal IgG] were immobilized to the plate surface and washed with phosphate-buffered saline prior to the addition of CD16^-^ NK cells (NK-92; open squares) or CD16^+^ NK cells (PTA-6967; filled squares). Following overnight incubation of the NK cells with plate-immobilized proteins, IFNγ secretion was by measured by ELISA; plotted as mean ± SEM (n=3) from a representative of two experiments. Tukey’s multiple comparisons tests (two-tailed) were performed for each protein between stimulated CD16^-^ NK cells (open squares) and stimulated CD16^+^ NK cells (filled squares). **: p < 0.01, ****: p < 0.0001.

**
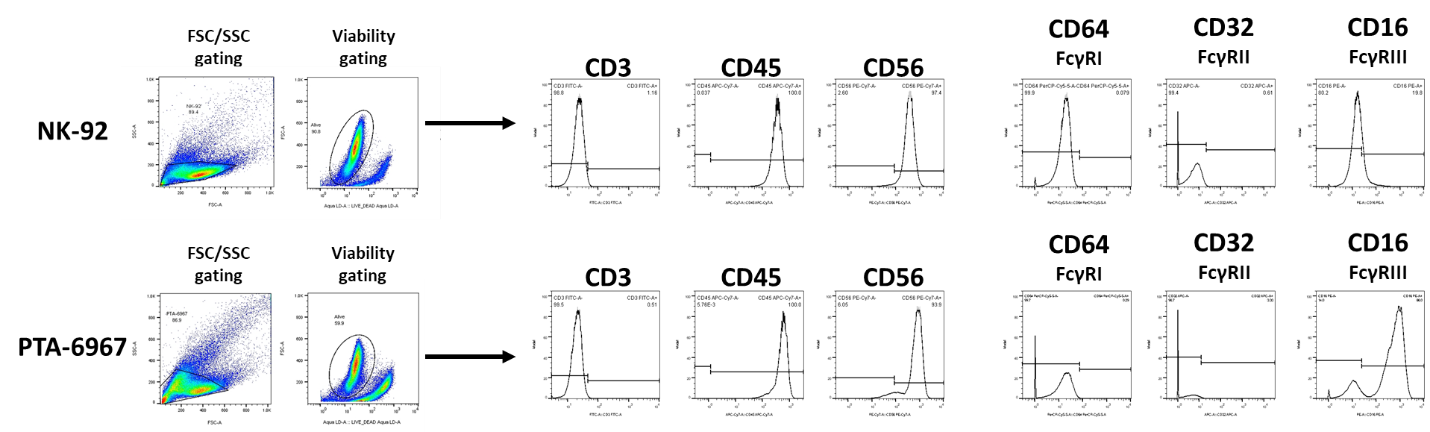
Supplementary Figure 4.** **Surface FcγR characterization of NK-92 and PTA-6967 NK cell lines**. NK-92 or PTA-6967 NK cell lines were viability stained with LIVE/DEAD Fixable Aqua Dead Cell Stain (405 nm excitation) in PBS and then surface stained with anti-CD3-FITC (BD# 561806) [Clone UCHT1], anti-CD16-PE (BD# 555407) [Clone 3G8], anti-CD64-PerCPCy5.5 (BD# 561194) [Clone 10.1], anti-CD56-PE-Cy7 (BD# 557747) [Clone B159], anti-CD32-APC (BD# 559769) [Clone FLI8.26], and anti-CD45-APC-H7 (BD# 560178) [Clone 2D1] in PBS + 1% BSA containing human Fc block. Stained cell samples were run on a BD Fortessa flow cytometer using positive and negative control beads for compensation. The percent positivity for each sample was calculated using fluorescence minus one (FMO) and unstained controls. Analyses were performed using FlowJo software (version 10). Pseudo-color dot plots depict the gating strategy, while histogram plots depict surface marker staining compared to FMO controls (bisector gate).

**
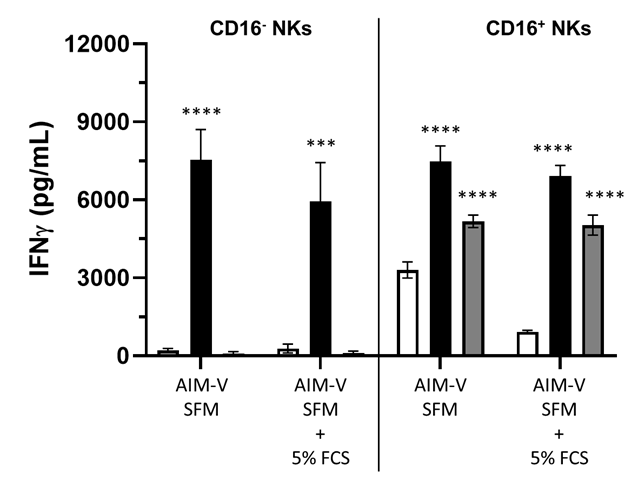
**

**Supplementary Figure 5.** **rFVIIIFc activates CD16^+^ NK cells in the absence of VWF.** IFNγ secretion by CD16^-^ NK cells (NK-92) or CD16^+^ NK cells (PTA-6967) measured by ELISA following overnight incubation with rFVIIIFc or PMA/ionomycin. NK cell *in vitro* stimulation conducted as described in Materials and Methods, except assay buffer NK cell media was replaced with AIM-V serum free medium or AIM-V serum free medium + 5% FCS (media alone, open bars; PMA/ionomycin, black bars; rFVIIIFc, grey bars); plotted as mean ± SEM (n=6) from two independent experiments. Student’s t tests (two-tailed) were performed between unstimulated cells (open bars) and rFVIIIFc or PMA/ionomycin stimulated cells (filled bars). ***: p < 0.001, ****: p < 0.0001.

**
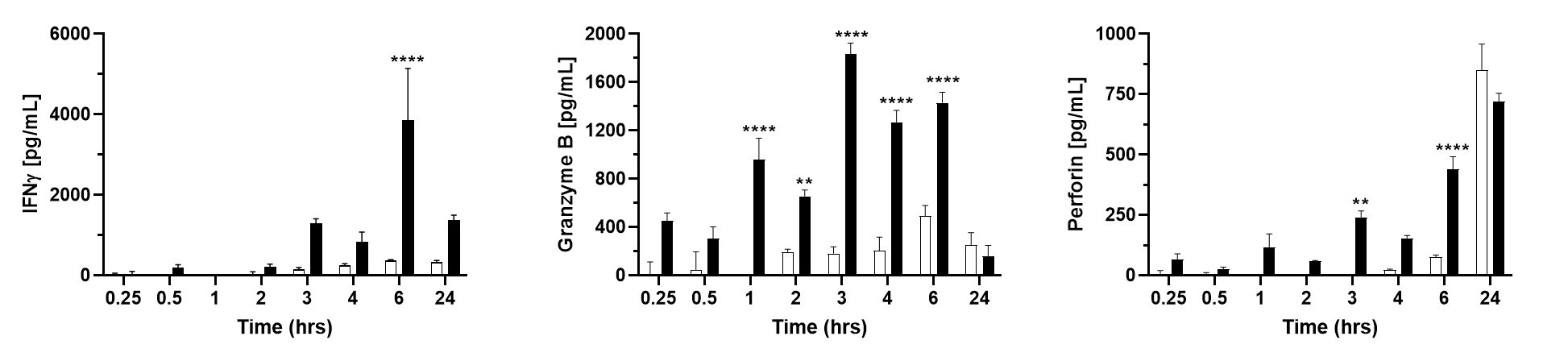
**

**Supplementary Figure 6.** **Dynamics of PMA/ionomycin stimulation on CD16^+^ NK cells.** Measurement of secreted IFNγ (left), granzyme B (middle), and perforin (right) by ELISA over time from PMA/ionomycin-stimulated CD16^+^ NK cells (PTA-6967); plotted as mean ± SEM (n=3). Tukey’s multiple comparisons tests (two-tailed) were performed at each timepoint between unstimulated cells (open bars) and rFVIIIFc stimulated cells (filled bars). **: p < 0.01, ****: p < 0.0001.

**
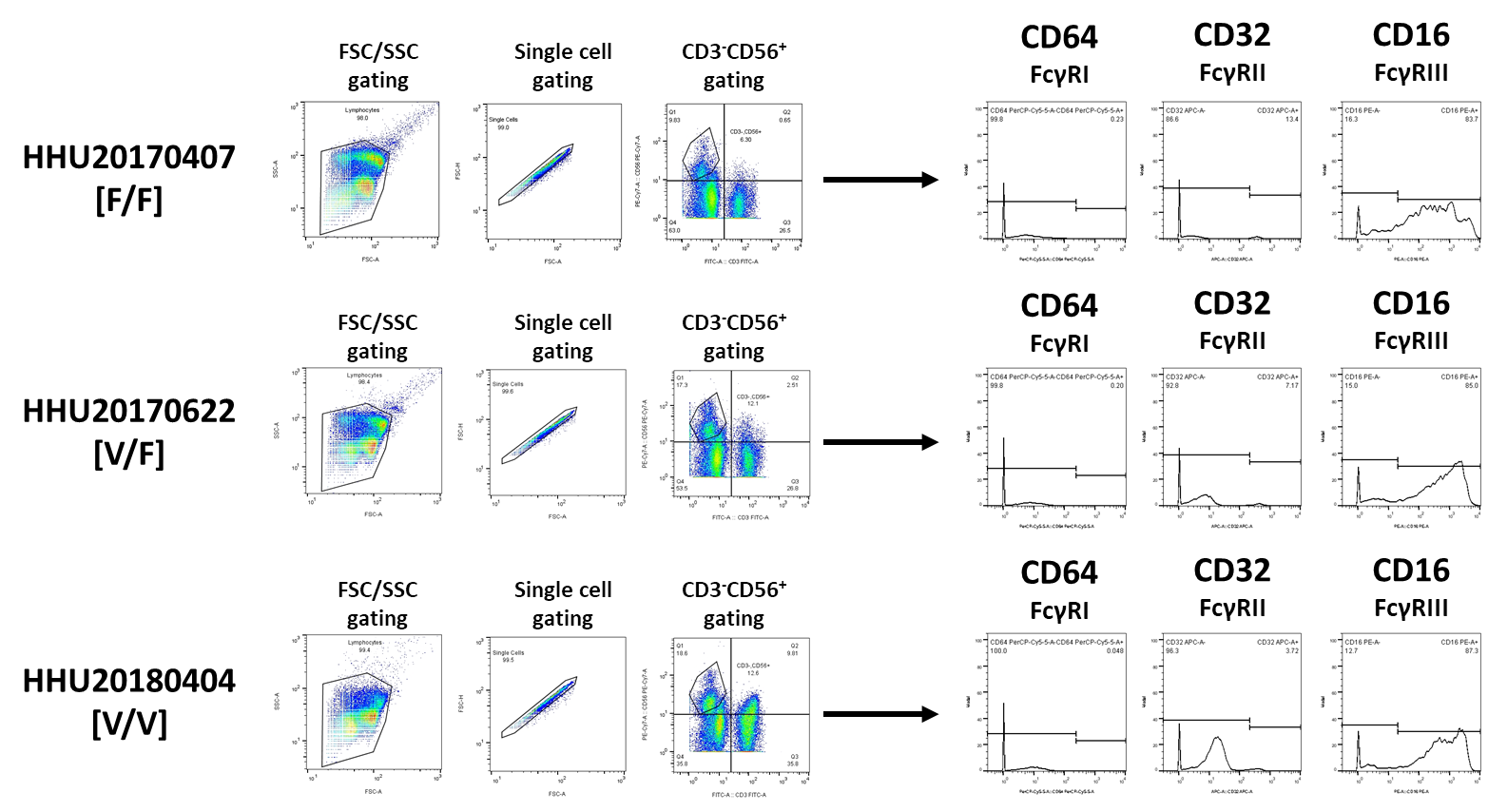
Supplementary Figure 7.** **Surface FcγR characterization of isolated primary human NK cells**. Human NK cells were isolated from PBMCs using Miltenyi NK Cell Isolation Kit (Miltenyi #130-092-657) with MS Columns (Miltenyi #130-042-201), and a MiniMACS Separator (Miltenyi #130-042-102). Following isolation, NK cell fractions were viability stained with LIVE/DEAD Fixable Aqua Dead Cell Stain (405 nm excitation) in PBS and then surface stained with anti-CD3-FITC (BD# 561806) [Clone UCHT1], anti-CD16-PE (BD# 555407) [Clone 3G8], anti-CD64-PerCPCy5.5 (BD# 561194) [Clone 10.1], anti-CD56-PE-Cy7 (BD# 557747) [Clone B159], anti-CD32-APC (BD# 559769) [Clone FLI8.26], and anti-CD45-APC-H7 (BD# 560178) [Clone 2D1] in PBS + 1% BSA containing human Fc block. Stained cell samples were run on a BD Fortessa flow cytometer using positive and negative control beads for compensation. The percent positivity for each sample was calculated using fluorescence minus one (FMO) and unstained controls. Analyses were performed using FlowJo software (version 10). Pseudo-color dot plots depict the gating strategy, while histogram plots depict surface marker staining compared to FMO controls (bisector gate).

**
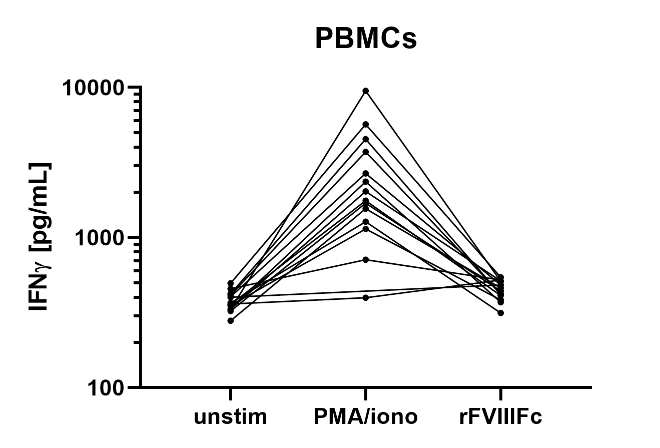

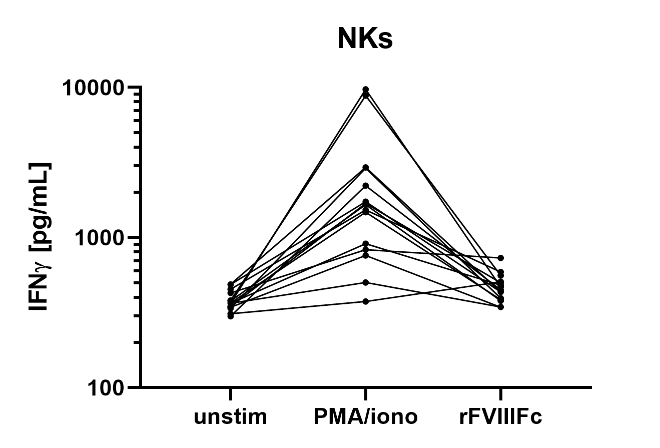
Supplementary Figure 8.** **Confirmation of IFNγ secretion by primary human PBMCs and isolated NK cells using CD16-independent PMA/ionomycin stimulation.** IFNγ secretion by primary PBMCs (left) and isolated NK cells (right) from 15 healthy human donors measured by ELISA following overnight incubation with PMA (50 ng/mL) and ionomycin (1 µg/mL), rFVIIIFc protein (250 nM) or media alone controls (unstimulated); plotted as IFNγ [pg/mL] with paired observations from each donor’s cells connected with a solid line.

**
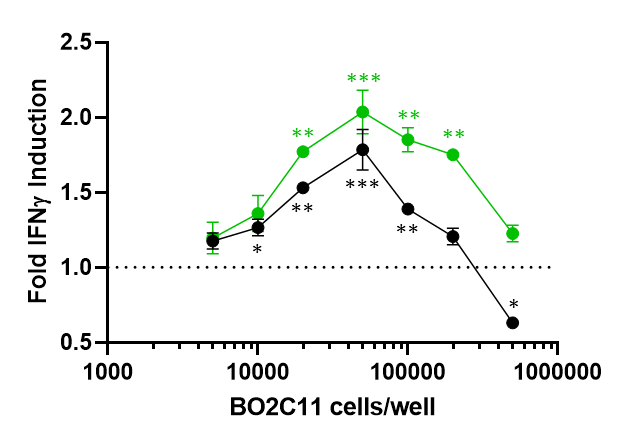
**

**Supplementary Figure 9.** **BO2C11-mediated enhancement of rFVIIIFc-induced IFNγ secretion from CD16^+^ NK cells (PTA-6967).** IFNγ secretion by CD16^+^ NK cells (PTA-6967; 100,000 cells/well) measured by ELISA following overnight incubation with 67 nM rFVIIIFc protein (black circle) or rFVIIIFc DP (green circle) in the presence of BO2C11 B cells [5,000 – 500,000 cells/well]; plotted as fold induction compared to no BO2C11 controls, mean ± SEM (n=2) from a representative of two experiments. Tukey’s multiple comparisons tests (two-tailed) were performed at each BO2C11 cell addition compared to no BO2C11 controls. *: p< 0.05, **: p < 0.01, ***: p < 0.001.
